# Supplementary figures and images for: International trade market forecasting and decision-making system: multimodal data fusion under meta-learning
Source: PeerJ Comput Sci. 2025 Aug 20;11:e3120. doi: 10.7717/peerj-cs.3120 (PMC12453693; doi:10.7717/peerj-cs.3120)

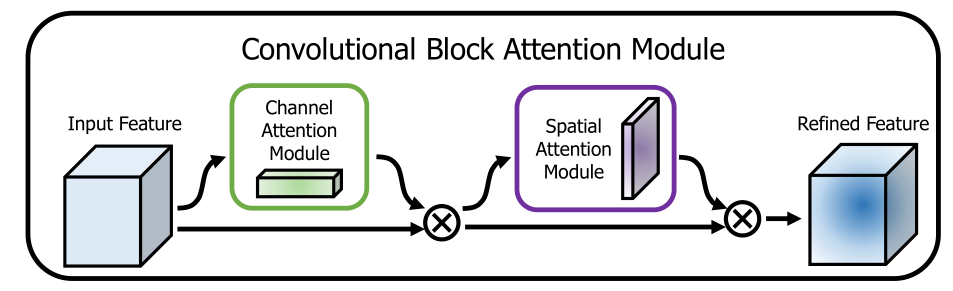

Supplement: Supplemental Information 1 [file peerj-cs-11-3120-s001.zip › code/imgs/01.png]
